# Structural and functional characterization of *DNAH5* variants in a Portuguese family with primary ciliary dyskinesia

**DOI:** 10.1007/s10815-026-03930-1

**Published:** 2026-06-19

**Authors:** Leonor Roseta, Maria João Oliveira, Eduarda Tinoco, Edite Ferreira, Inês Sanches, Regina Monteiro, Ivone Pascoal, Joana Saranago, Telma Oliveira, Rute Pereira, Jorge Oliveira, Luís Gales, João Freixo, Rosália Sá, Mário Sousa

**Affiliations:** 1 Pulmonary Rehabilitation Unit, Department of Pulmonology, Local Health Unit of Gaia-Espinho, Vila Nova de Gaia, Portugal; 2 UMIB-Unit for Multidisciplinary Research in Biomedicine/ITR-Laboratory for Integrative and Translational Research in Population Health, University of Porto, Porto, Portugal; 3 Department of Otorhinolaryngology, Local Health Unit of Gaia-Espinho, Vila Nova de Gaia, Portugal; 4 Laboratory of Cell Biology, Department of Microscopy, ICBAS-School of Medicine and Biomedical Sciences, University of Porto, Porto, Portugal; 5 Agência Nacional de Inovação, Porto, Portugal; 6 Center for Predictive and Preventive Genetics (CGPP), IBMC-Institute of Cellular and Molecular Biology, i3S-Institute of Health Research and Innovation, University of Porto, Porto, Portugal; 7 Department of Chemistry, ICBAS-School of Medicine and Biomedical Sciences, University of Porto, Porto, Portugal; 8 Centro Hospitalar Universitário de São João, Unidade Local de Saúde de São João, Porto, Portugal

**Keywords:** Primary ciliary dyskinesia, *DNAH5* variants of unknown significance, High-speed videomicroscopy, Transmission electron microscopy, Genetic screening, Immunofluorescence

## Abstract

**Purpose:**

Primary ciliary dyskinesia (PCD) is a rare genetic heterogeneous disorder mainly characterized by impaired mucociliar clearance and chronic respiratory symptoms. Although *DNAH5* is commonly implicated in PCD, several *DNAH5* variants remain unclassified.

**Methods:**

The proband was studied by high-speed videomicroscopy, transmission electron microscopy, whole exome sequencing and immunofluorescence. Protein structural analysis was performed through structural models obtained by X-ray crystallography and cryo-electron microscopy. The family was studied by Sanger sequencing of the variants, high-speed videomicroscopy and immunofluorescence.

**Results:**

The patient carry, in heterozygosity, the *DNAH5* c.5290 T > C p.(Ser1764Pro) missense variant of uncertain significance and the pathogenic truncated variant *DNAH5* c.4237C > T p.(Gln1413*). He was clinically diagnosed in adulthood with PCD, confirmed following nasal nitric oxide measurement, high-speed videomicroscopy and transmission electron microscopy, all of which revealed hallmark PCD defects, with decreased nasal nitric oxide levels (30 nl/min) and ciliary beating frequency (0.66 Hz), a dyskinetic ciliary beating pattern (62.5% total immotility) and a class-1 ultrastructure. Immunofluorescence analysis demonstrated reduced *DNAH5* expression, and protein structural models predicted that the variant of uncertain significance causes an unstable protein. Family analyses confirmed a trans-inheritance and uncovered a brother with a similar PCD phenotype, the same two variants and similar reduced *DNAH5* expression.

**Conclusions:**

Results support a pathogenic role for the c.5290 T > C p.(Ser1764Pro) variant and elucidate the effects of the other variant. These results underscore the importance of integrating clinical, ultrastructural, molecular and protein expression analyses to clarify and contribute to PCD diagnosis, besides now serving as potential markers for diagnostics and targeted therapies.

**Supplementary Information:**

The online version contains supplementary material available at https://doi.org/10.1007/s10815-026-03930-1.

## Introduction

Primary ciliary dyskinesia (PCD, ORPHA:244) is a rare phenotypical and genetically heterogeneous autosomal recessive disorder [1], being rarely autosomal dominant [2] or X-linked [3], whose genetic consequences affect cilia motility [4]. PCD occurs in approximately 1:15,000–1:30,000 live births (Orphanet database), but this estimate is uncertain as, due to its clinic heterogeneity and absence of a gold-standard diagnostic test, the disease is prone to misdiagnosis or underdiagnosis [5]. This situation is due to the fact that as cilia proteins are numerous, there is also the possibility of generating several pathogenic variants, which explains the heterogeneous phenotypes and the lack of a single diagnostic tool.

Motile cilia beat rhythmically [6], being present in the respiratory tract [1], ependyma [7], fetal node [8], Fallopian tubes [9] and endometrium [10]; the spermatozoon flagellum shares with cilia the same motility apparatus, the axoneme [11]. As a consequence, an abnormal axoneme function, caused by mutations in genes associated to the biogenesis and structure of motile cilia and flagellum proteins [12], will affect mucociliar clearance, laterality, fluid flow (hydrocephalus), embryo transport (Fallopian tube), implantation (endometrium) and sperm motility [13].

PCD usually manifests in early childhood with persistent wet cough, chronic nasal congestion, recurrent otitis media, and often unexplained neonatal respiratory distress. Approximately half of the patients exhibit laterality defects, and a subset presents the classical triad of situs inversus, chronic sinusitis and bronchiectasis, defining the Kartagener syndrome [14–16]. In PCD context, male infertility has been observed in about 81% of the cases [17], female infertility in 61% of the cases [17, 18], in which, besides impaired Fallopian tube embryo transfer, was also related to interference with implantation due to the interaction of the blastocyst with endometrium cilia [10], and development of hydrocephalus in 6% of the cases [2, 19].

For managing PCD diagnosis, the guidelines of the European Respiratory Society and of the American Thoracic Society recommend a stepwise diagnostic approach integrating clinical features and specialized testing [20]. Nasal nitric oxide is a non-invasive screening tool that, when persistently low, supports the diagnosis, but requires additional confirmatory testing; high-speed videomicroscopy gives cilia frequency and pattern of motion information; and transmission electron microscopy assess ultrastructural defects that can be diagnostic [14, 20, 21]. Genetic testing has become central to PCD diagnosis and biallelic pathogenic variants in one of over 50 known disease-causing genes confirm the diagnosis; however, the frequent identification of variants of uncertain significance (VUS) remains a major limitation [14]. Immunofluorescence has recently emerged as a valuable adjunct, enabling the detection of decreased, absent or mislocalized ciliary proteins. This is useful when transmission electron microscopy results are inconclusive and may aid in VUS interpretation, which continues to pose a significant challenge [15].

The Dynein Axonemal Heavy Chain-5 gene (*DNAH5*) encodes a dynein protein, which is part of the axoneme outer dynein arms (ODA), a microtubule-associated motor protein complex consisting of heavy, light and intermediate chains. Protein *DNAH5* (4624 amino acids) functions as a force-generating protein with ATPase activity, whereby the release of ADP is thought to produce the force-producing power stroke. Mutations in this gene cause PCD type-3, as well as Kartagener syndrome. Gene *DNAH5* (chromosome 5p15.2; 79 exons) is one of the most frequently implicated genes in PCD, being linked to absence of the ODA [22], and with several VUS reported in databases.

We report a male fertile patient with clinical PCD that carry in heterozygosity two *DNAH5* variants, one missense variant c.5290 T > C p.(Ser1764Pro), which remains unclassified, along with a pathogenic truncated variant c.4237C > T p.(Gln1413*), which remains to be functionally characterized. Escorted by ultrastructural, structural and functional characterization of the variants, and family studies, we proved the pathogenicity of the variants and uncovered an infertile brother with the same two variants. Results indicate the importance of integrating clinical, molecular and functional data in PCD diagnosis, and supported these variants to be included in diagnostic panels, futurizing targeted therapies.

## Materials and methods

### Ethical considerations

All procedures were conducted in accordance with applicable ethical standards and institutional protocols. Patient records were reviewed in accordance with confidentiality protocols to obtain comprehensive data, including demographic characteristics, clinical and microbiological data, pulmonary function, imaging studies, as well as diagnostic exams. All participants provided written informed consent for the collection and use of both clinical data and biological material for research purposes, in accordance with the approval granted by the Joint Ethics Committee of the Hospital and University, CHUP/ICBAS approval number 2020–094 (077-DEFI-078-CE). This work did not involve human or animal experiments and thus the provisions of the Declaration of Helsinki as revised in Tokyo 2004 do not apply to this work.

### Patient

The patient of the present study presented a PCD phenotype, and the genetic study revealed two heterozygotic variants in the *DNAH5* gene, the missense *DNAH5* c.5290 T > C p.(Ser1764Pro) VUS and the pathogenic truncated variant *DNAH5* c.4237C > T p.(Gln1413*). During family studies, the same two variants were observed in his brother.

### Nasal sample collection

Nasal cells were obtained by nasal brushing from the proband and family, using a cytology soft sterile brush (Endobrush, Biogyn SNC, Mirandola, Italy), in both nostrils [23, 24]. After brushing, cells of one nostril were placed in Medium-199 (Gibco, ThermoFisher Scientific, USA), supplemented with 50 μg/mL PenStrep, for high-speed video microscopy analysis and immunofluorescence. Cells from the other nostril were placed in fixative (2.5% glutaraldehyde in cacodylate buffer 0.1 M) for transmission electron microscopy analysis. Family cells were not used for transmission electron microscopy analysis.

### Ciliary beat frequency and beat patterns by high‑speed video microscopy

Ciliary beat frequency and ciliary beat patterns were evaluated as previously described, with some adaptations [25]. Briefly, collected ciliated cells were placed in Medium-199, and immediately upon arrival at the laboratory, placed at 37 °C until analysis. Analysis was conducted in an inverted microscope (Olympus, Nikon, Tokyo, Japan). Undisrupted ciliated clusters devoid of mucus were selected for study. Beating ciliated cells were recorded using a digital high-speed video camera (Semiconductor Vita 5000, Pixelink/Navitar, Inc., New York, USA) at a rate of 300 to 400 frames per second. The ciliary beat frequency was analyzed using the semi-automated method with the CiliarMove program [26]. In total, 14 × 3 and 20 × 3 ciliary regions, from proband and parents respectively, were examined. To assess ciliary beat patterns, each edge was analyzed using ImageJ, version 1.53e [27]. Coordinated ciliary beat in a back-and-forth movement along the entire epithelial edge was defined as normal. The dyskinetic beat pattern was categorized into distinct ciliary beat patterns by a modification of previously reported descriptions [28, 29].

### Transmission electron microscopy procedure

Nasal samples of the proband were analyzed as previously reported [23, 24]. Briefly, samples were fixed with 2.5% glutaraldehyde (Sigma-Aldrich, Missouri, USA) in 0.1 M cacodylate buffer (Merck, Darmstadt, Germany), pH 7.2, 2 h, room temperature (RT), post-fixed with 2% osmium tetroxide (Merck) in buffer, 2 h, 4 °C, and dehydrated in a graded ethanol series (VWR, Pennsylvania, USA), then treated with 1% tannic acid (Merck) in 100% ethanol and embedded in epoxy resin (Epon, Sigma-Aldrich). Semithin and ultrathin sections were cut on an LKB-ultramicrotome (Leica Microsystems, Wetzlar, Germany), using diamond knives (Diatome, Pennsylvania, USA). Suitable areas of ciliated cells were selected in semithin Sects. (1 μm), and stained with methylene blue-Azur II (Merck). Ultrathin sections were retrieved on copper grids (Taab, Berks, England). After double-contrasting with aqueous uranyl acetate (BDH, Poole, England) and lead citrate (Merck), they were observed and photographed on a JEOL 100CXII transmission electron microscope (JEOL, Tokyo, Japan), operated at 60 kV.

### Quantitative transmission electron microscopy morphologic analysis determination of classes

Cilia axoneme ultrastructure was evaluated at high magnifications in transverse sections. Diagnosis was based on the presence of a systematic defect in any of the axonemal structures [30], according to the quantitative methods provided by the international consensus guideline for reporting transmission electron microscopy results in the diagnosis of PCD (BEAT PCD TEM Criteria) [31]. In these criteria, the axoneme structure is presented in three distinct morphological classes, class-1 defects (indicative of PCD), class-2 defects (suggestive of PCD) and class-normal (normal axoneme structure), requiring the analysis of at least 50 axonemes in high magnification cross-sections that show complete ciliary membrane and intact ciliary structure.

Class-1 defects confirm the diagnosis of PCD, and include defects in ODA [presence of ≥ 5 outer dynein arm defects (absence, shorter, badly defined) in > 50% axonemal cross sections], combined defects in ODA [presence of ≥ 5 outer dynein arm defects in > 50% axonemal cross sections] and IDA [presence of ≥ 7 inner dynein arm defects (absence, shorter, badly defined) in > 50% axonemal cross sections], and disorganization of axonemal microtubules with absence of IDA [disruption of the 9 + 2 symmetry in > 25% axonemal cross sections combined with > 7 inner dynein arm defects in > 50% axonemal cross sections]. Class-2 defects only indicate a diagnosis of PCD when associated with other complementary tests (high-speed videomicroscopy, immunofluorescence or genetics), and include number of ciliated cells (decreased if < 50% in semithin sections cross sections), central pair defects (absence, altered number, displaced), basal body mislocalization (displacement or misalignment of basal bodies regarding the apical membrane of ciliated cells in relation to longitudinally well-orientated cilia axonemes) (Supplementary Fig. 1), disorganization of axonemal microtubules with absence of IDA [disruption of the 9 + 2 symmetry in > 25% axonemal cross sections combined with > 7 inner dynein arm defects in > 50% axonemal cross sections], defects in ODA [presence of ≥ 5 outer dynein arm defects in 25–50% axonemal cross sections], and combined defects in ODA [presence of ≥ 5 outer dynein arm defects in 25–50% axonemal cross sections] and IDA [presence of ≥ 7 inner dynein arm defects (absence, shorter, badly defined) in 25–50% axonemal cross sections] [31].

### Quantitative transmission electron microscopy ciliary beat axis analysis

The ciliary beat axis and the ciliary deviation were evaluated in a minimum of 100 transverse sections examined (Supplementary Fig. 2). In selected images, a line was drawn parallel to the central microtubules of each cross section. Based on the main orientation of the drawn lines, a reference line was then drawn. This reference line is perpendicular to the other lines and passes between the two microtubules of the central pair of a chosen cilium. The angle of each line to the reference line is then calculated. Angles are then subtracted from their mean. The mean of the subtracted angles is equal or lower than zero. The standard deviation (SD) of the subtracted angles corresponds to the ciliary beat axis and ciliary deviation [32]. In these criteria, cilia movement orientation is presented as three distinct types, PCD-type (cilia orientation compatible with the distribution described in patients with ciliary movement disorder such as primary ciliary dyskinesia), BQ-type (cilia orientation compatible with the distribution described in patients with ciliary movement disorder such as bronchiectasis) and Normal-type (cilia orientation compatible with the distribution described in healthy patients).

### Quantitative transmission electron microscopy determination of the inflammation degree

In a minimum of 200 cross Sects. (612 in the present case), cilia were quantified regarding the presence of inflammation signs, which included blebs (membrane protrusions, enlargement of the cytoplasmic space), compound cilia (several axonemes in the same cilium), naked cilia (absence or poor definition of the cilium membrane, with three degrees of severity: < 25%, 25–50%, and > 50%) and total axoneme microtubule disarrangement [33, 34].

### Nucleic acid extraction

Patient (proband), both parents and brother peripheral blood was collected in EDTA tubes (VACUETTE, Porto, Portugal). Genomic DNA was extracted from peripheral blood leukocytes, using the QIAsymphony system designed for automated purification from human whole blood.

### Whole-exome sequencing

Whole-exome sequencing was performed using Agilent’s SureSelect Human All Exon V6 capture kit performed on NovaSeq 6000 (Illumina) system. Variant analysis was restricted a virtual multigene panel of 50 genes associated with primary ciliary dyskinesia and differential diagnosis): *ARMC4*, *CFAP300* (*C11ORF70*), *CFAP298* (*C21ORF59*), *DNAAF19* (*CCDC103*), *ODAD1* (*CCDC114*), *CCDC39*, *CCDC40*, *CCDC65*, *CCNO*, *CENPF*, *CFAP57* (*WDR65*), *CFTR*, *DNAAF1*, *DNAAF2*, *DNAAF3*, *DNAAF5* (*HEATR2*), *DNAAF6* (*PIH1D3*), *DNAAF11* (*LRRC6*), *DNAH1*, *DNAH11*, *DNAH5*, *DNAH8*, *DNAH9*, *DNAI1*, *DNAI2*, *DNAJB13*, *DNAL1*, *DRC1* (*CCDC164*), *DNAAF4* (*DYX1C1*), *GAS2L2*, *GAS8*, *HYDIN*, *INVS*, *LRRC56*, *LZTFL1*, *MCIDAS* (*IDAS*), *NEK10*, *ODAD3* (*CCDC151*), *OFD1*, *RPGR*, *RSPH1*, *RSPH3*, *RSPH4A*, *RSPH9*, *SPAG1*, *STK36*, *NME8* (*TXNDC3*), and *ZMYND10*. Variants were filtered by their minor allele-frequency (MAF below 1% in population databases: NCBI’s dbSNP, 1000 Genome Project, Exome Variant Project, ExAC and gnomAD), for those that resulted in a change at the protein level and/or previously described in NCBI’s ClinVar.

### Sanger sequencing

To perform variant segregation analysis in the family, specific primers were designed to amplify the genomic regions encompassing the variants identified in the proband: *DNAH5*_Ex27_F: 5′-TGTAAAACGACGGCCAGTCATACATCTCTGGCTTGCTTGT-3′, *DNAH5*_Ex27_R: 5′-CAGGAAACAGCTATGACCGACAAGAACAATGCAGCAAGAT-3′; and *DNAH5*_Ex33_F: 5′-TGTAAAACGACGGCCAGTATTTTGTATTGCCTTTTAGATTTCA-3′, *DNAH5*_Ex33_R: 5′-CAGGAAACAGCTATGACCATACAATGTGATCAACAGCTGTG-3′. After PCR amplification products were purified with Exo/SAP Go (GRiSP, Portugal) and sequenced by Sanger sequencing, using Big Dye Terminator Cycle Sequencing v1.1 (Thermo Fisher Scientific, USA) analyzed on an ABI 3500 Dx Genetic Analyzer (Thermo Fisher Scientific, USA). Sequencing results were analyzed in SeqScape software version v3.0 (Thermo Fisher Scientific, USA).

### Immunofluorescence

Immunofluorescence analysis of nasal epithelial cells was performed as previously described [12, 24, 35]. Briefly, cell suspensions were spread onto glass slides (LABSOLUTE, Renningen, Germany), air dried, and stored at − 80 °C until use. Cells were fixed with 4% paraformaldehyde (20 min, RT) (Merck) in PBS pH 7.4 (PAN-Biotech, Germany), permeabilized with 0.2% Triton X-100 (Sigma-Aldrich) (15 min, RT) in PBS, and blocked with 5% non-fat milk (60 min, RT) (Nestlé, Vevey, Switzerland). Cells were then incubated overnight at 4 °C with primary antibody rabbit anti-*DNAH5* (HPA037470; Atlas Antibodies, Stockholm, Sweden) (for outer dynein arms), rabbit anti-DNALI1 (HPA028305: Atlas Antibodies, Stockholm, Sweden) (for inner dynein arms), rabbit anti-GAS8 (HPA041311; Atlas Antibodies, Stockholm, Sweden) (for nexin-dynein regulatory complexes), rabbit anti-RSPH9 (HPA031703; Atlas Antibodies, Stockholm, Sweden) (for radial spokes), and mouse anti-acetylated α-tubulin (6-11B-1; Santa Cruz Biotechnology, Texas, USA) (for axonemal microtubules). For each experiment, a negative control, through the omission of the primary antibody, was included. Goat anti-rabbit IgG (H + L) cross-adsorbed secondary antibody, Alexa Fluor™ 488: A-11008, and CoraLite594-conjugated goat anti-mouse IgG(H + L) (Proteintech) were used as secondary antibodies. Cells were counterstained with Vectashield mounting medium containing 4′,6-diamidino-2-phenylindole (DAPI: Vector Laboratories, California, USA). Results were observed in an epifluorescence microscope (Eclipse E400; Nikon, Tokyo, Japan). The corrected total cell fluorescence (CTCF) was calculated, according to the formula CTCF = integrated density − (selected cell area × mean fluorescence of background readings). Statistical analysis was performed with the GraphPad Prism software, applying the Mann–Whitney test, with significance levels at alpha < 0.05.

### Protein structural analysis

Structural models obtained by X-ray crystallography and cryo-electron microscopy from multiple sources and available in the Protein Data Bank were analyzed [36]. These included models of human and *DNAH5* dynein axonemal heavy chains [37], as well as non-human axonal structures. Analysis of the Ser1764Pro mutation was based on the human *DNAH5* model (PDB ID: 8J07) [38] and pairwise sequence alignment with the 380-kDa motor domain of the *Dictyostelium discoideum* cytoplasmic dynein model (PDB ID: 3VKG) [36]. Representations of the models were prepared using PyMOL (Schrödinger, L. The PyMOL Molecular Graphics System version 3.1. https://www.scirp.org/reference/ReferencesPapers?ReferenceID=1571978) and ChimeraX [39].

## Results

**Fig. 1:**
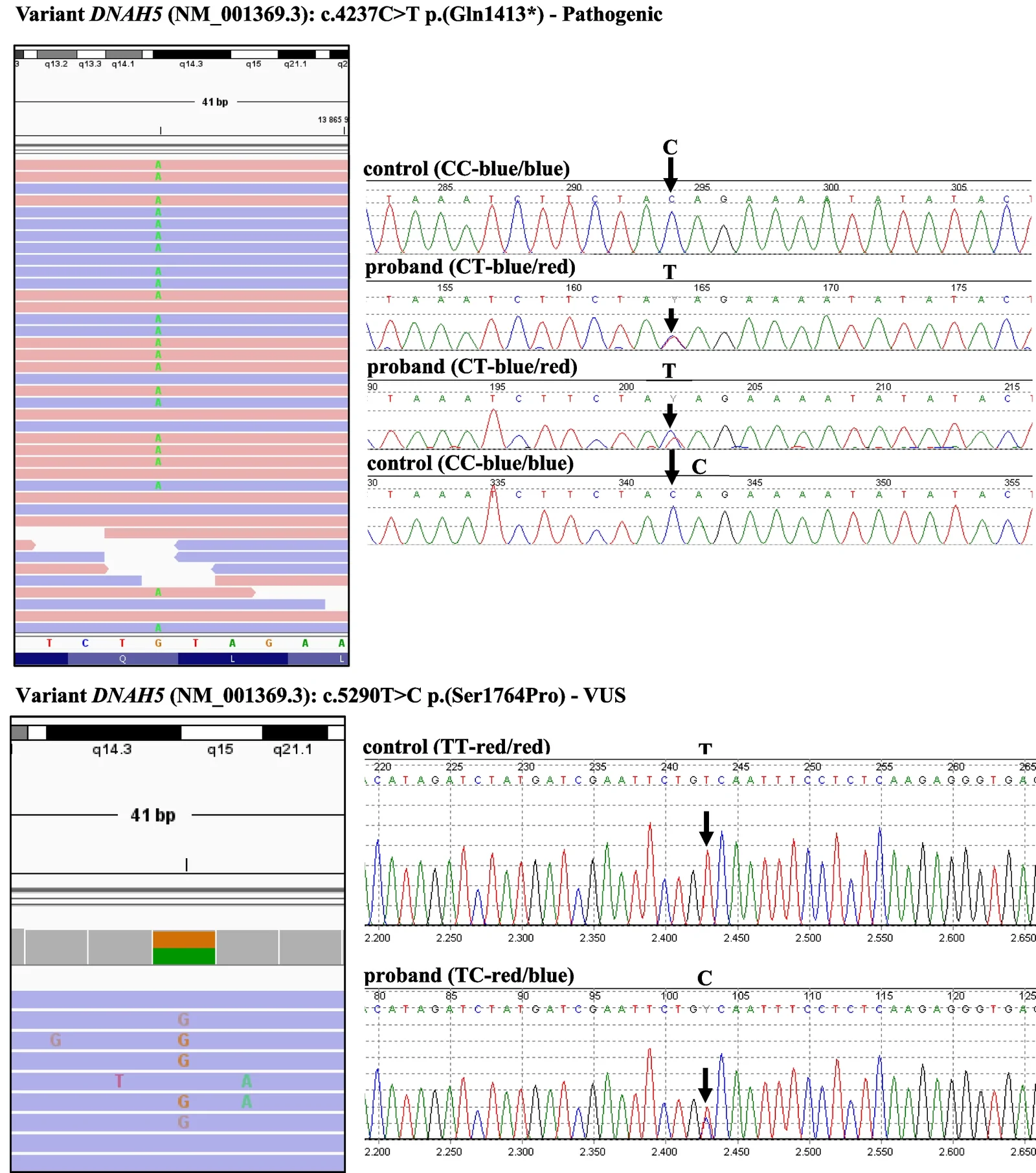
Whole-exome sequencing reads alignments (left side) and Sanger sequencing electropherograms (right side) of the variants found in the proband. The nonsense variant *DNAH5* NM_001369.3:c.4237C > T [p.(Gln1413*)] was found in heterozygosity. The normal alleles in controls reveal the presence of a cytosine at position c.4237 (CC-blue/blue), whereas the proband evidences a substitution at the same position of the cytosine by a thymidine (CT-blue/red). The missense variant *DNAH5* NM_001369.3:c.5290 T > C [p.(Ser1764Pro)] was also found in heterozygosity. The normal alleles in controls reveal the presence of a thymine at position c.5290 (TT-red/red), whereas the proband evidences a substitution at the same position of the thymidine by a cytosine (TC-red/blue)

**Fig. 2:**
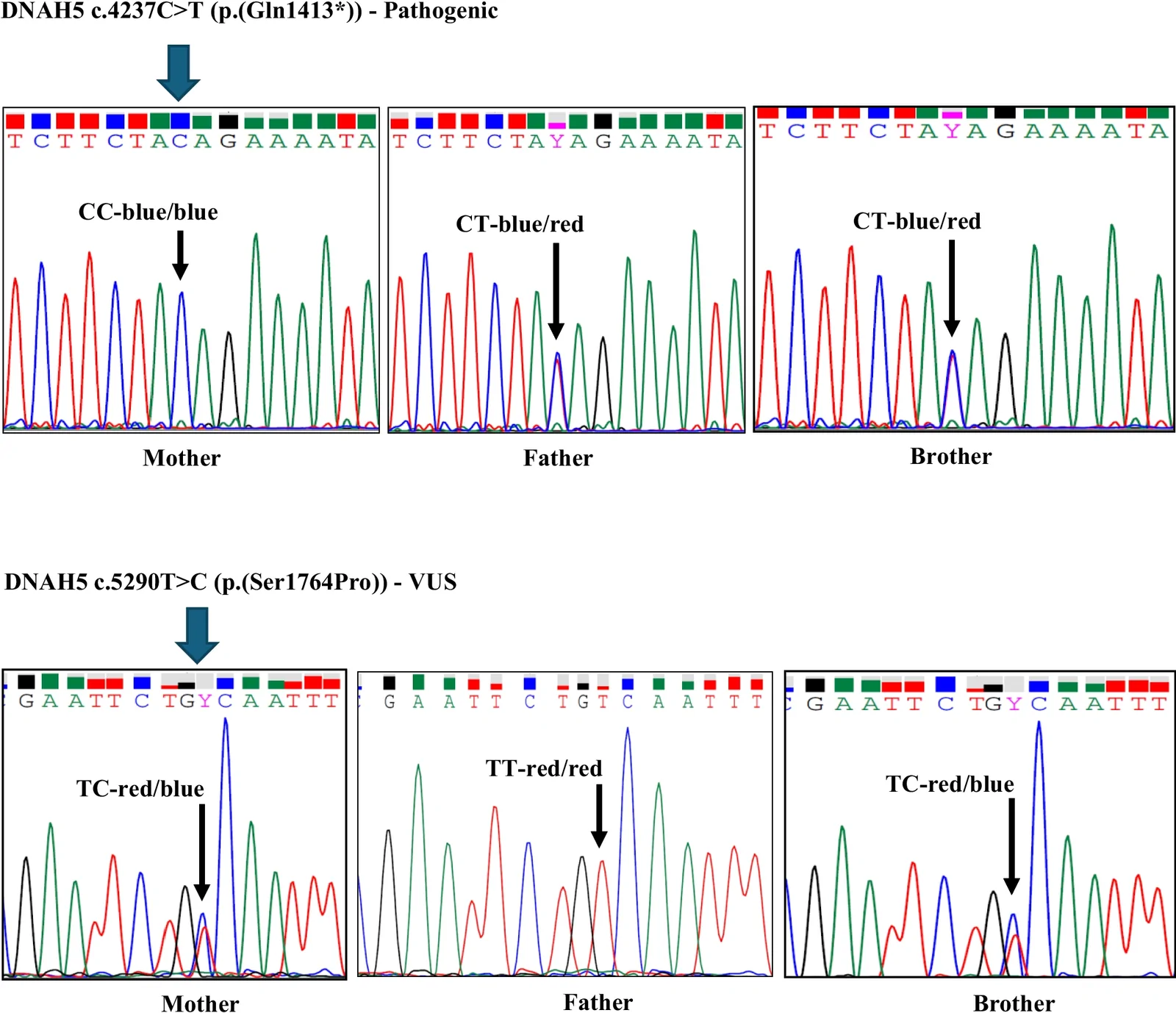
Sanger sequencing electropherograms of the variants found in the family. The nonsense variant *DNAH5* NM_001369.3:c.4237C > T p.(Gln1413*) was found in heterozygosity in the father and brother. The normal alleles in controls reveal the presence of a cytosine at position c.4237 (CC-blue/blue), whereas the affected relatives evidence a substitution at the same position of the cytosine by a thymidine (CT-blue/red). The missense variant *DNAH5* NM_001369.3:c.5290 T > C p.(Ser1764Pro) was also found in heterozygosity in the mother and brother. The normal alleles in controls reveal the presence of a thymine at position c.5290 (TT-red/red), whereas the proband evidences a substitution at the same position of the thymidine by a cytosine (TC-red/blue)

**Fig. 3:**
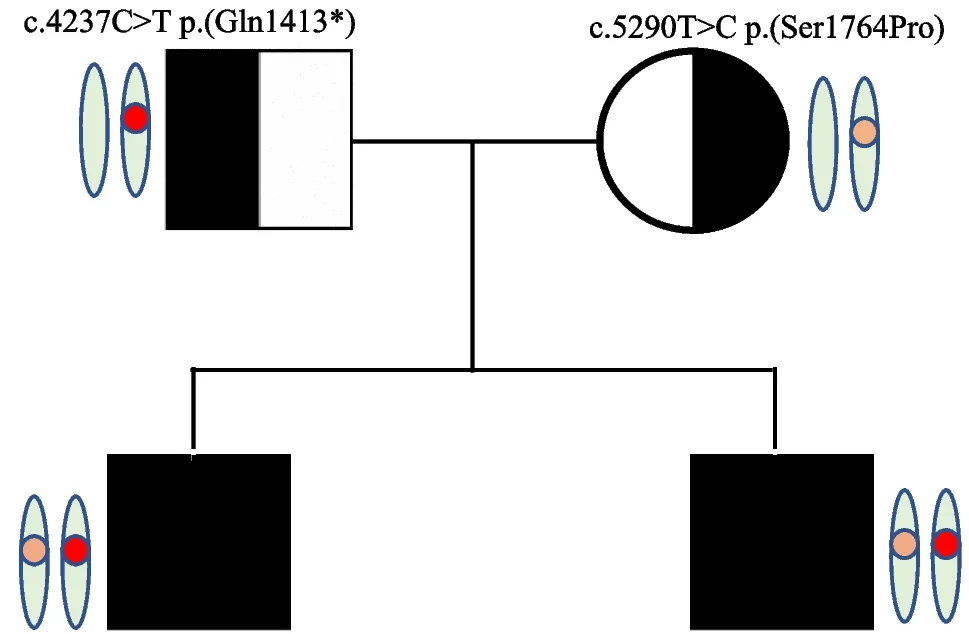
Genogram. The pathogenic truncated variant *DNAH5* (NM_001369.3): c.4237C > T p.(Gln1413*) was found in the father and the missense variant of unknown significance *DNAH5* (NM_001369.3): c.5290 T > C p.(Ser1764Pro) was found in the mother, at different alleles. The proband and his brother present both variants in heterozygosity. Data confirms the presence of trans inheritance and thus of a compound heterozygosity in both brothers

The patient evidenced two heterozygotic variants in the *DNAH5* gene, the missense *DNAH5* c.5290 T > C p.(Ser1764Pro) variant of unknown significance and the pathogenic truncated variant *DNAH5* c.4237C > T p.(Gln1413*), detected after Whole-exome sequencing (Fig. 1). Sanger sequencing of first-degree family members (Fig. 2) revealed that the truncated variant was inherited from the father and that the VUS was inherited from the mother, whereas the brother presented both variants also in heterozygosity, establishing a compound heterozygosity (Fig. 3).

**Fig. 4:**
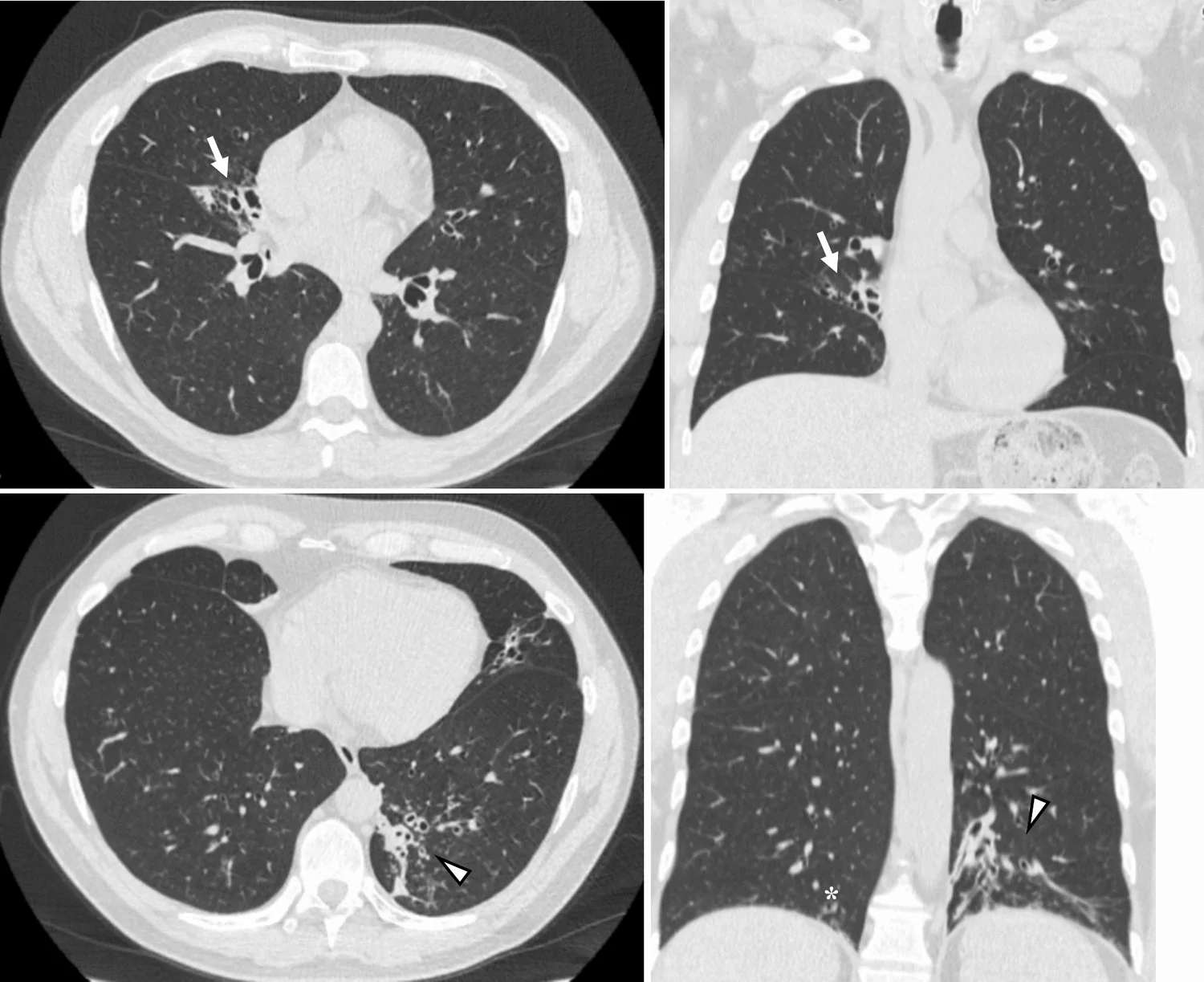
High-resolution chest computed tomography of the proband. Cylindrical and varicose bronchiectasis and endobronchial plugs are predominantly observed in the right middle lobe (arrows) and in the posterior-basal segments of the left lower lobe (arrowheads). Additionally, tree-in-bud micronodules (*) are present in the middle-right lobe and bilaterally in the lower lobes, suggestive of ongoing inflammatory/infectious processes with endobronchial dissemination

The patient was 44 years old at diagnosis and exhibited since childhood daily productive cough and recurrent respiratory tract infections, requiring repeated antibiotic therapy. He presents bronchorrhea, chronic rhinosinusitis and nasal polyposis, the last requiring surgery. High-resolution computed tomography of the chest demonstrated cylindrical and varicose bronchiectasis involving the middle lobe and right lower lobe, characterized by diffuse thickening of the bronchial walls and mucous impaction (Fig. 4). The nasal nitric oxide measurement using a stationary chemiluminescence analyzer during velum closure, yielded a low value of 30 nL/min. Lung functional tests were normal. Alpha-1-antitrypsin assay, immunological studies, and atopy tests were normal. Cystic fibrosis was excluded based on a normal sweat chloride test. The patient has been treated with long-term azithromycin for frequent exacerbations, with no specific pathogen identified in sputum. The PICADAR score [40] revealed a value of 3, and the patient did not present laterality defects nor infertility (spontaneous conception with delivery of two healthy children).

During family study, the patient’s 48-year-old brother was reported to be under follow-up at another hospital for chronic rhinosinusitis, nasal polyposis (requiring surgical intervention), and bronchiectasis. He did not display laterality defects but presented infertility due to oligoasthenoteratozoospermia, requiring intracytoplasmic sperm injection to obtain the desired child (healthy female of 14 years old). The proband’s father (73 years old) and mother (72 years old) evidenced no respiratory complaints, and the marriage was not consanguineous.

**Fig. 5:**
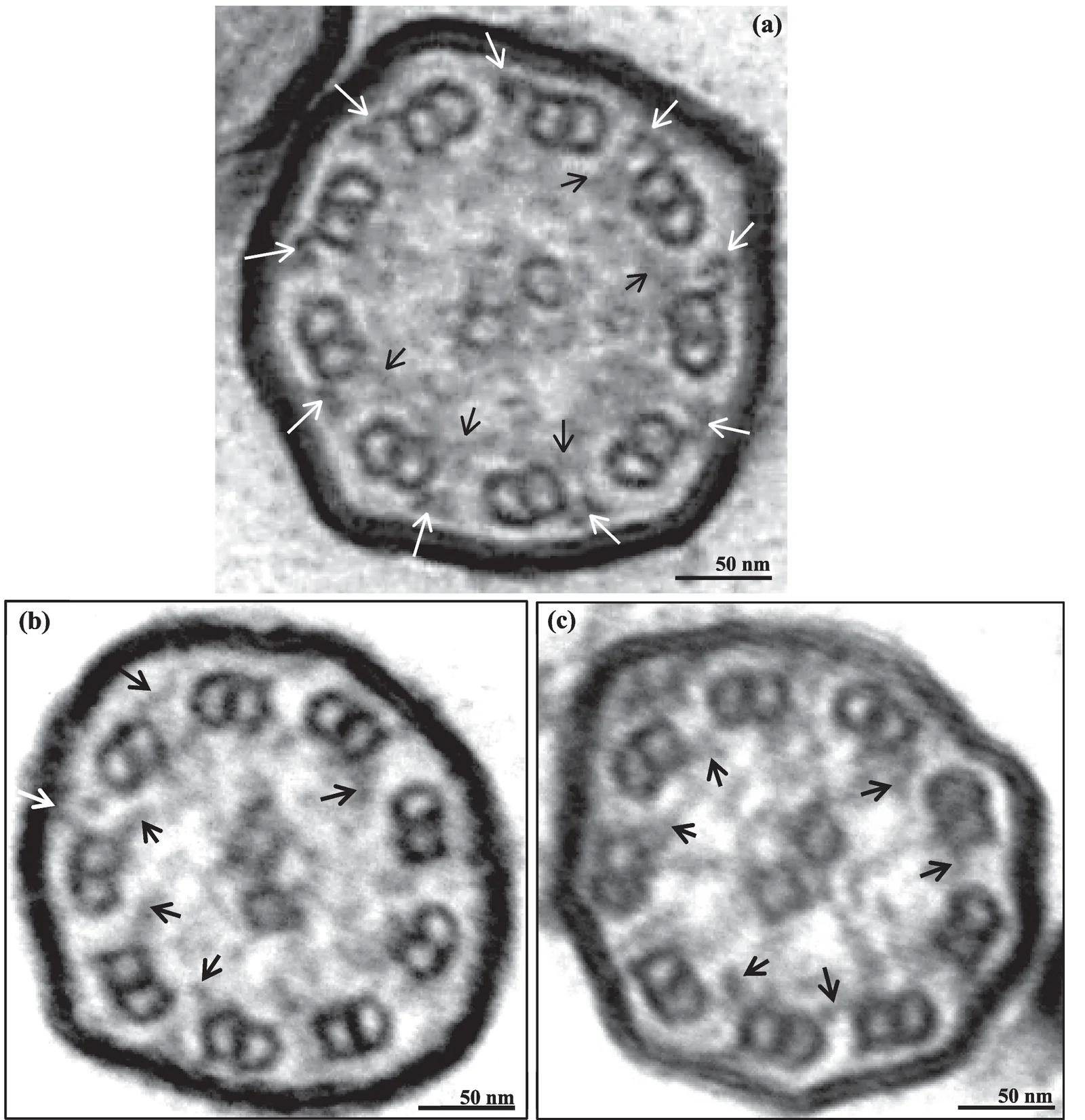
Ultrastructural images of the axoneme from nasal ciliated cells of the proband. Arrows indicate dynein arms that serve as a template for this axoneme. According to international guidelines a normal axoneme must contain at least 5 clearly visible outer dynein arms (ODA) and 3 inner dynein arms (IDA), with inner dynein arms frequently being less defined. **a** Normal axoneme from another case (the present case did not have normal axonemes) with absent, decreased size, or poorly defined ODA (*n* = 0, pathological ≥ 5) and with absent, decreased size, or poorly defined IDA (*n* = 4 pathological ≥ 7). **b** Case with absent, decreased size, or poorly defined ODA (*n* = 7, pathological ≥ 5) and with absent, decreased size, or poorly defined IDA (*n* = 5, pathological ≥ 7). **c** Case with absent, decreased size, or poorly defined ODA (*n* = 9, pathological ≥ 5) and with absent, decreased size, or poorly defined IDA (*n* = 3, pathological ≥ 7)

**Fig. 6:**
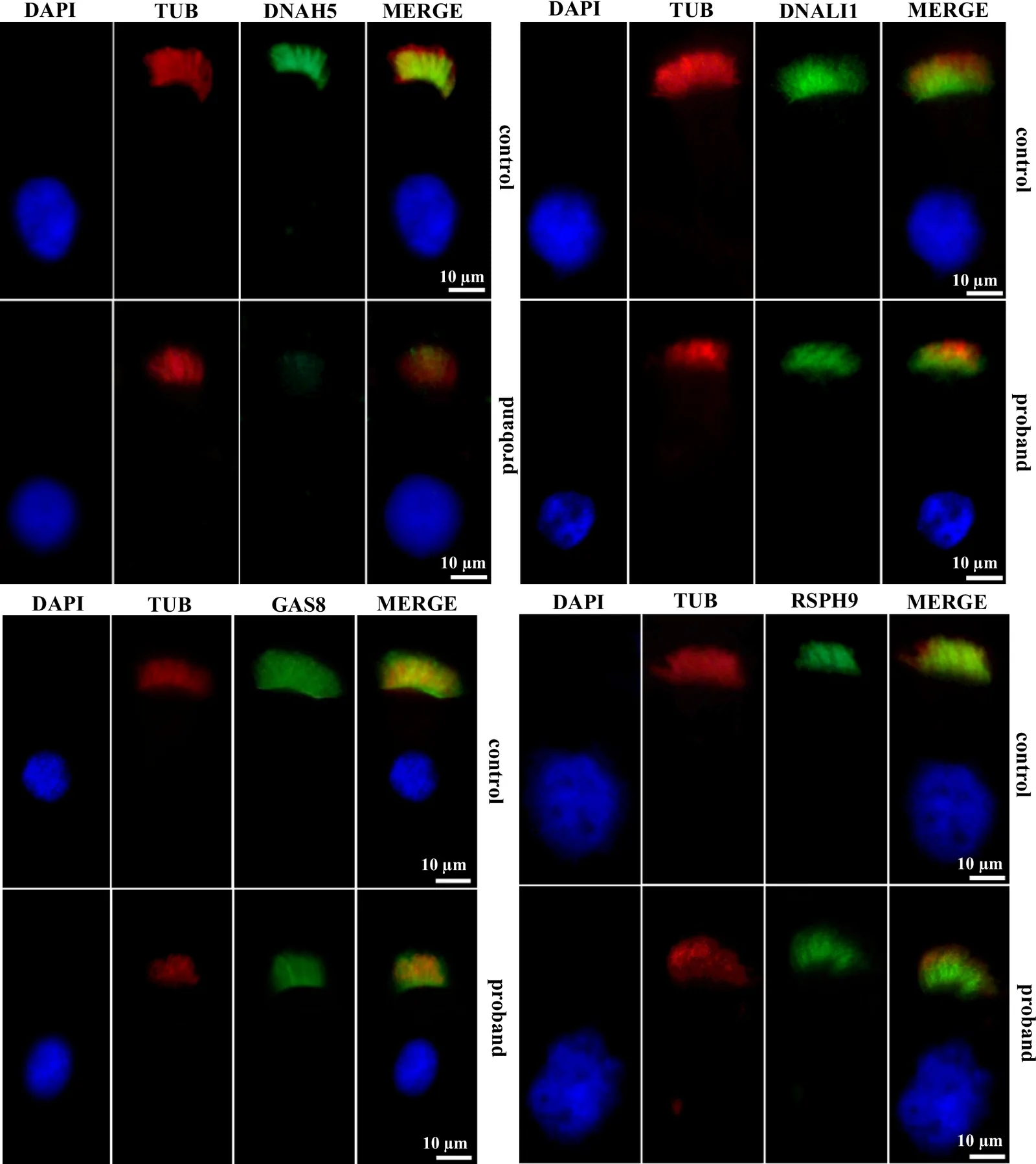
Immunofluorescence analysis of respiratory epithelial cells from the Proband. Each panel shows staining for a different axonemal protein: *DNAH5*, component of the outer dynein arms (ODA); DNALI1, component of the inner dynein arms (IDA); GAS8, component of the nexin–dynein regulatory complex (NDRC); and RSPH9, component of the radial spokes (RS). Cells were stained with DAPI (blue, nuclei), anti-acetylated tubulin (TUB, red, ciliary axonemes), and antibodies against the corresponding axonemal protein (green). In all panels, the Merge images showed co-localization. Fluorescence localization of DNALI1, GAS8 and RSPH9 (were preserved and comparable to expected control patterns. In contrast, the *DNAH5* signal was markedly reduced

**Fig. 7:**
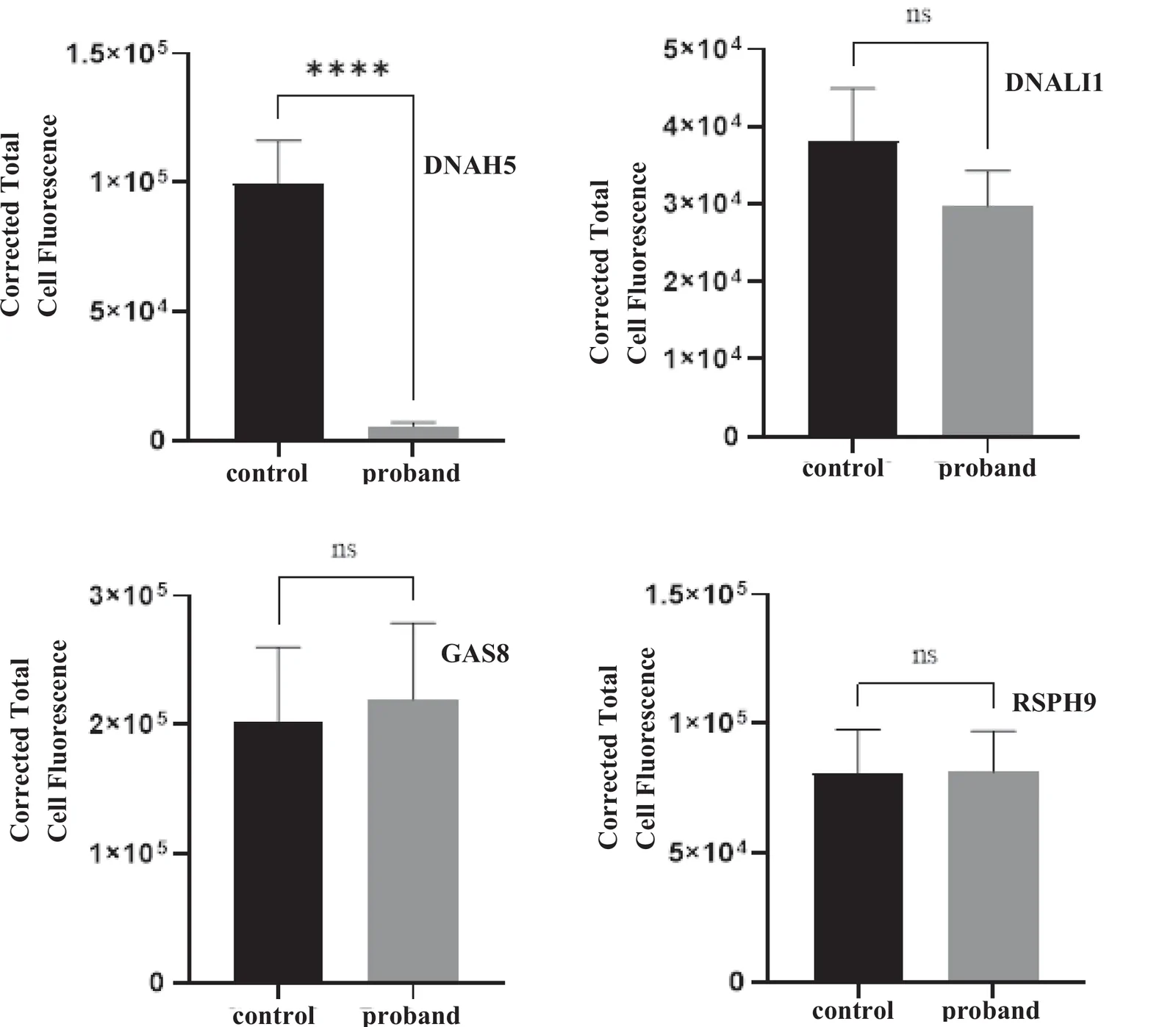
Statistical analysis of the mean fluorescence intensity of axoneme proteins, using the statistical test Kruskal–Wallis for multiple comparisons, taken from the immunofluorescence analysis of the Proband respiratory epithelial cells. Corrected total cell fluorescence was calculated from the measurements obtained from the ImageJ software. Statistical significance was set at an alpha value of < 0.05. Fluorescence intensity of DNALI1 (for inner dynein arms), GAS8 (for nexin–dynein regulatory complex) and RSPH9 (for radial spokes) antibodies were preserved and comparable to expected control patterns. In contrast, *DNAH5* (for outer dynein arms) fluorescence intensity was markedly reduced. This reduction was statistically significant (*P* < 0.0001), based on the comparison of mean fluorescence intensity in control versus the Proband samples, and is consistent with selective loss of outer dynein arms components

In the proband, high-speedvideomicroscopy analysis revealed a markedly reduced ciliary beat frequency mean (0.66 Hz; range, 0.0–5.8 Hz; reference, 12.75 Hz; range, 7.0–19.0 Hz) and a fully dyskinetic ciliary beat pattern, with 62.5% of the immotile type and 37.5% of the partially immotile type. Quantitative transmission electron microscopy studies showed 26.3% of inflammatory signs, 58.8% of misaligned basal bodies, no central pair defects, 26.9% of ciliary beat axis deviation, and 97% of cilia axonemal abnormalities, with 66.7% showing class-1 defects affecting the outer dynein arms in 97% of the cross sections (Fig. 5). Genetic testing identified two heterozygous *DNAH5* variants: c.4237C > T p.(Gln1413*), classified as pathogenic (truncated), and c.5290 T > C p.(Ser1764Pro), classified as VUS (missense) (Fig. 1). To further investigate the impact of the identified variants, immunofluorescence was performed on nasal ciliated cells, which revealed a significant reduction in *DNAH5* expression along the axoneme and an overall decrease in fluorescence intensity, supporting the pathogenic relevance of the *DNAH5* defect (Figs. 6 and 7).

**Fig. 8:**
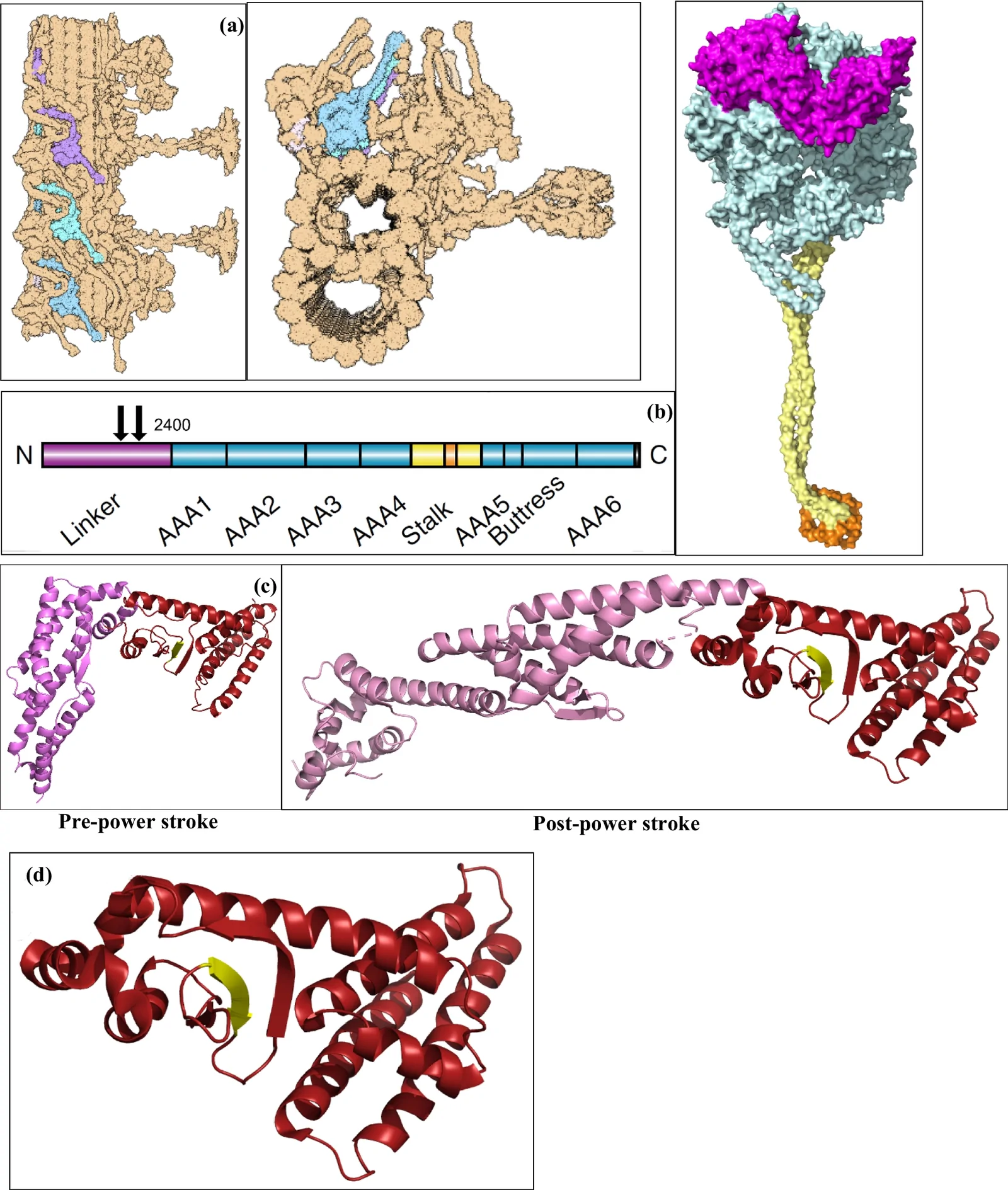
Structural models obtained by X-ray crystallography and cryo-electron microscopy of protein *DNAH5*. **a** Orthogonal views of the 96-nm repeat of the human respiratory doublet microtubule and associated axonemal complexes, including *DNAH5* (highlighted in the figure; structural model PDB ID: 8J07 [38]. **b** Schematic representation of a dynein motor domain. Structural elements are color-coded: the N-terminal linker, the AAA + domains (AAA1–AAA6) responsible for ATP binding and hydrolysis, and the coiled-coil stalk terminating in the MTBD. The truncation site (Gln1413) and the missense site (Ser1764) are located in the N-terminal linker, as indicated by arrows. The truncated protein, if expressed, would lack essential domains, resulting in loss of function. **c** The linker is subdivided into four regions: the mobile Link1–2 subdomain (purple), which interacts variably with the AAA1 ring across dynein structures, and the static Link3–4 subdomain (red). During the power-stroke priming step, Link1–2 undergoes a rigid-body movement relative to Link3–4. **d** The Ser1764Pro mutation is located within the middle strand (highlighted in yellow) of a three-stranded antiparallel β-sheet in Link3–4. Substitution with proline disrupts the β-sheet hydrogen-bonding network, likely reducing the stability and rigidity of the Link3–4 region. (Pre-power-stroke structure: PDB ID 4RH7 [37]; post-power-stroke structure: PDB ID 3VKG [36]

Structural model analysis of the *DNAH5* protein (Fig. 8) revealed that the truncation site and the missense site are located in the N-terminal Linker. Regarding the truncated variant c.4237C > T p.(Gln1413*), this sequence change creates a premature translational stop signal (p.Gln1413*) in the *DNAH5* gene (exon-27). It is expected to result in an absent or disrupted protein product, as loss-of-function variants in *DNAH5* are known to be pathogenic; if the truncated protein would be expressed, it would lose essential domains (all after position 1413), resulting in loss of function (non-functional protein). The Linker region of *DNAH5* is composed of a mobile Link1–2 domain (α-conformation) and a static Link3–4 domain (α-conformation). During the power-stroke priming step, Link1–2 undergoes a rigid-body movement relative to Link3–4. The missense VUS c.5290 T > C p.(Ser1764Pro) is located in the coding exon-33 of the *DNAH5* gene, and results from a T to C substitution at nucleotide position 5290. The serine at codon 1764 is replaced by a proline residue, located within the middle strand of the three-stranded β-sheet in the Link3–4 region. This substitution introduces a conformational constraint, as the cyclic proline side chain restricts backbone flexibility and disrupts the regular hydrogen-bonding pattern characteristic of β-strands. Consequently, the mutation is expected to locally destabilize the β-sheet architecture, potentially reducing the overall stability and rigidity of the Link3–4 region (Fig. 8).

**Fig. 9:**
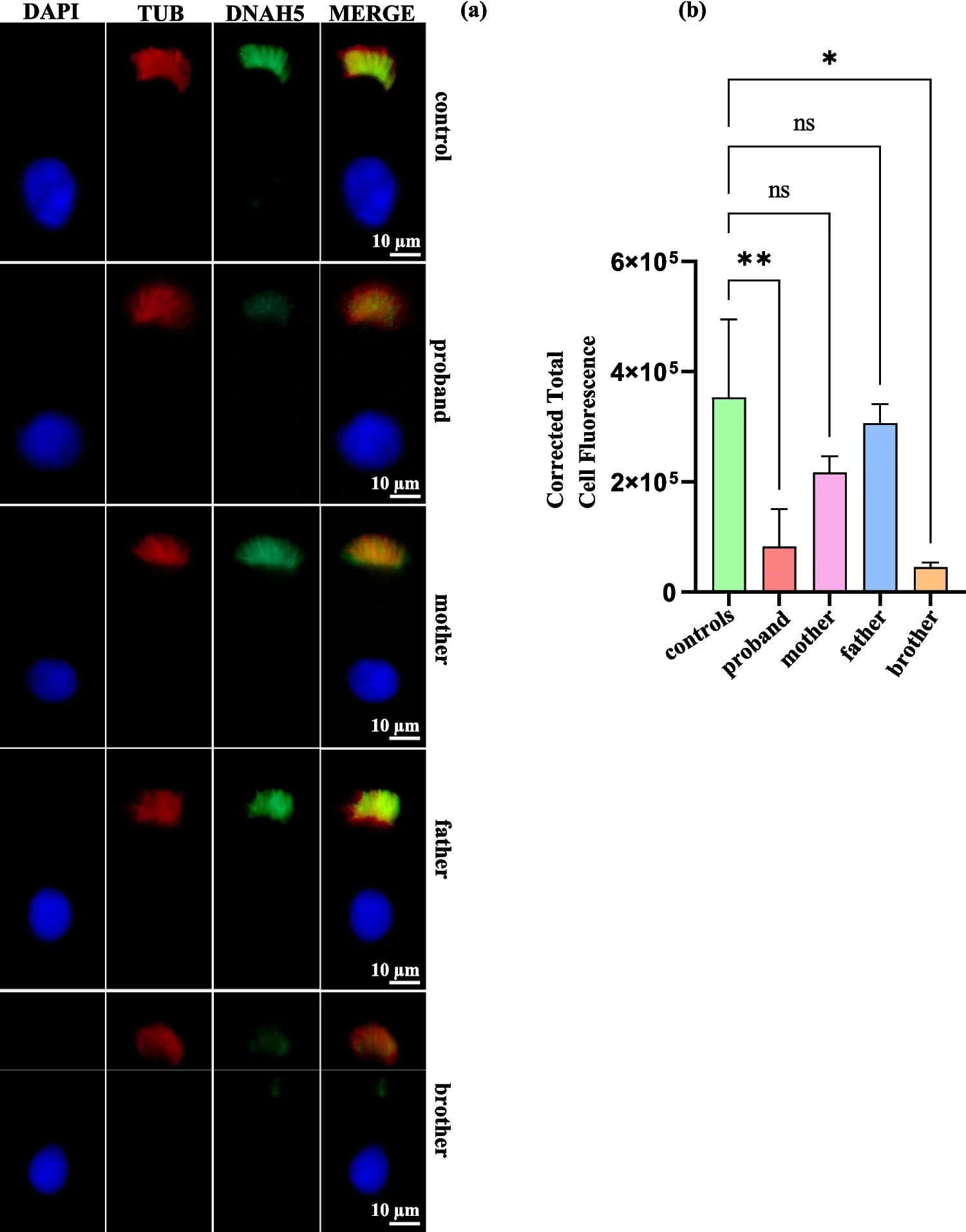
Immunofluorescence analysis of respiratory epithelial cells from the Proband and family members. **a** Cells were stained with DAPI (blue, nuclei), anti-acetylated tubulin (TUB, red, ciliary axonemes), and the axonemal protein *DNAH5* (green). In all panels, the Merge images showed co-localization. Fluorescence localization of *DNAH5* was preserved and comparable to expected control patterns in the mother and father. In contrast, the *DNAH5* (for outer dynein arms) signal was markedly reduced in both the Proband and brother. **b** Statistical analysis of the mean fluorescence intensity of axoneme proteins, using the statistical test Kruskal–Wallis for multiple comparisons, taken from the immunofluorescence analysis of the Proband and family members respiratory epithelial cells. Corrected total cell fluorescence was calculated from the measurements obtained from the ImageJ software. Statistical significance was set at an alpha value of < 0.05. Fluorescence intensity of *DNAH5* (for outer dynein arms) was markedly reduced in both the Proband and his brother. This reduction was statistically significant (*P* < 0.0001), based on the comparison of mean fluorescence intensity in control versus the Proband and his brother samples, and is consistent with selective loss of outer dynein arms components

The genetic study of family members revealed that the father carried the pathogenic variant and the mother carried the VUS, confirming a compound heterozygosity, whereas the brother carried both variants also in heterozygosity (Figs. 2 and 3). Although proband parents did not present protein expression abnormalities (Fig. 9), ciliary beat frequency values were significantly decreased (father, 5.59 Hz; 3.71–7.94; mother, 6.53 Hz; 2.65–10.06), and the majority of the ciliary movements were dyskinetic (father, 76.9%; 73.1% of the low-amplitude coordinated type and 3.8% of the coordinated and partially immotile type, with 23.1% of the normal type; mother, 72.7% of the low-amplitude coordinated type, with 27.3% of the normal type). The proband brother presented decreased ciliary beat frequency (mean, 0.51 Hz; range, 0.0–3.18) and a ciliary beat pattern 100% dyskinetic (57.5% of the immotile type and 42.5% of the coordinated and partially immotile type), and markedly reduced *DNAH5* expression (Fig. 9).

## Discussion

In this report, we describe two brothers from a non-consanguineous family, which presented a PCD phenotype and both carrying the same two *DNAH5* variants in heterozygosity, the c.5290 T > C p.(Ser1764Pro) missense variant, currently classified as VUS, and the truncated pathogenic variant c.4237C > T p.(Gln1413*), still remaining functionally uncharacterized.

Gene *DNAH5* (OMIM number, 608,644) encodes a dynein-heavy chain forming part of the axoneme outer dynein arms (ODA), which is essential for ciliary motility. Mutations in the gene *DNAH5* typically lead to absent or dysfunctional ODA, resulting in impaired mucociliar clearance and the characteristic clinical features of PCD [41]. Biallelic pathogenic variants in *DNAH5* account for a substantial proportion of genetically confirmed PCD cases [41, 42].

In the proband, the diagnosis of PCD was supported by the typical clinical features and multiple abnormal diagnostic tests, consistent with international guidelines. The diagnostic work-up revealed low levels of nasal nitric oxide, abnormal ciliary motion frequency and a predominant immotile pattern on high-speed videomicroscopy, and class-1 defects on transmission electron microscopy, specifically affecting the ODA. Additionally, immunofluorescence demonstrated markedly reduced *DNAH5* expression. The brother also presented the typical clinical features of PCD, whose diagnosis was confirmed by an abnormal ciliary motion frequency and a predominant immotile pattern on high-speed videomicroscopy, and reduced *DNAH5* expression in immunofluorescence analysis. The low PICADAR score observed in the proband may have contributed to late diagnosis in the 4th decade of life, His brother, as well, was also only diagnosed following the present investigation at the age of 48. The Sanger variant sequencing in the family showed segregation of the variants, strengthening the diagnostic conclusion.

The simultaneous presence of the two heterozygote variants here described, c.5290 T > C p.(Ser1764Pro) missense VUS and the truncated pathogenic variant c.4237C > T p.(Gln1413*), were previously found in two Canada emigrated Portuguese patients and in a Portuguese case. However, these were never functionally characterized. Of the emigrated cases, one was a female of 6 years old and the other a male of 14 years old. Both presented ODA defects, with the female exhibiting situs inversus, and with both parents having no consanguineous marriage. No further information was given [43, 44]. The Portuguese case corresponds to a male diagnosed at 8 years old [45], showing Kartagener syndrome. This patient (presently of 26 years old), exhibited a PICADAR score of 7, unexplained neonatal respiratory distress and bronchospasm, requiring hospitalization, and, since childhood, daily productive cough and recurrent respiratory tract infections requiring repeated antibiotic therapy. He developed chronic rhinosinusitis, nasal polyposis (requiring surgery), and bronchiectasis, identified in high-resolution computed tomography (cylindrical and varicose, in median and both inferior lobes). Lung function tests showed mild restrictive changes. Due to chronic Pseudomonas aeruginosa infection, he has been receiving inhaled colistimethate sodium. A recent semen analysis was facilitated, which revealed oligoasthenoteratozoospermia [45].

Regarding the c.5290 T > C p.(Ser1764Pro) missense variant, here identified in heterozygosity in the *DNAH5* gene, it is found in the gnomAD population database with an allele frequency of 2:128,522 (1:64,261), and in the ClinVar database there is no consensus regarding its classification (pathogenic; likely pathogenic; or of uncertain significance). Computational predictive methods (CADD, MutationTaster, and SIFT) are concordant regarding the functional impact of this variant. This alteration has been reported in four individuals with PCD (Supplementary Table 1). One patient presented the variant in homozygosity (French case), had ODA defects but no further information was given [46]. Another patient presented the variant in heterozygosity (British case), in association with two other *DNAH5* variants of uncertain significance, showing a PICADAR score of 8, a total immotility pattern by high-speed videomicroscopy and an ODA defect [47]. The third patient (Portuguese case), presented the variant in heterozygosity in association with the c.4237C > T p.(Gln1413*) variant, as in the present report, and presented situs inversus totalis, a PICADAR score of 7 and oligoasthenoteratozoospermia [45]. The fourth patient presented the same variant in heterozygosity in association with another variant in the *DNAH5* gene, and situs inversus totalis [48]. In comparison, the present case did not have laterality defects; nitric oxide levels were only here reported; only two cases reported PICADAR evaluation, being higher than the cutoff of 5, contrary to our score, which was normal; only one case performed high-speed videomicroscopy analysis, showing a null cilia beat frequency (almost null in our present case) and an immotility cilia beat pattern (our case presented 63% of immotility); two cases performed transmission electron microscopy analysis, and, as in the present case, it was found an ODA defect; variant consequences at the protein level as evaluated by immunofluorescence was performed only in the present study (loss of the protein in cilia); and the fertility status was revealed only in one case, being infertile, whereas our patient was fertile. The present combined expression studies in both the proband and family, family variant sequencing and structural protein analysis, now allowed the reclassification of variant *DNAH5* c.5290 T > C p.(Ser1764Pro) to a pathogenic missense variant.

In relation to the c.4237C > T p.(Gln1413*) truncated variant, identified in the present study as heterozygous in the *DNAH5* gene, it is found in the gnomAD population database with an allele frequency of 2:128,530 (1:64,265) in the European population, and is classified as pathogenic in the ClinVar database (1 entry). This alteration has been previously reported (HGMD: CM137177) in Portuguese patients with PCD [43–45], and leads to the insertion of a premature stop codon; therefore, it can be expected that a truncated protein will be produced or that no protein will be produced due to transcript degradation mediated by nonsense variants. With this information, the variant was classified [49] as pathogenic. Three reports previously presented results on the c.4237C > T p.(Gln1413*) truncated variant (Supplementary Table 1). Of the patients reported by Kim et al. [43] and Marshall et al. [44], one had situs ambiguous (6 years, female) and the other situs solitus (14 years, male), and of the cases presented by Tinoco et al. [45], one had situs inversus totalis (8 years, male) and the other (30 years, male) presented no laterality defects, as in our case. In no case there was family consanguinity, and all cases presented heterozygosity. In all cases, the nitric oxide levels were low (< 77 nl/min). The PICADAR score was above the cutoff of 5, except in the present case. Curiously, our case had 44 years at diagnosis, suggesting that lower PICADAR score may be expected with later diagnosis. Besides the present case, highspeed videomicroscopy was applied only in one previous patient [45], with both showing significantly decreased cilia beat frequency, and a significantly increased cilia immotility pattern. Transmission electron microscopy analysis was also performed in the two patients presented by Kim et al. [43] and Marshall et al. [44], and in one patient of those presented by Tinoco et al. [45], with results showing a significant outer dynein arm defect, as in the present case. In relation to functional studies, protein spatial localization and intensity detected by immunofluorescence was first reported here, demonstrating that these variants are associated with a severe loss of cilia *DNAH5*. Only two cases reported the fertility status, curiously with the same two variants, one being fertile (present report) and the other with oligoasthenoteratozoospermia [45]. Family segregation was only presented here. The present combined expression studies in both the proband and family, family variant sequencing and structural protein analysis now enabled to confirm the pathogenicity of the variant.

The presence in both brothers of the *DNAH5* truncate variant in one allele and of the *DNAH5* missense variant in the other allele explain the decrease in cilia beating frequency and the cilia dyskinetic beating pattern, the predominant absence of the outer dynein arms, as well as the marked reduction in protein cilia localization. Each variant was found independently in parents, which evidenced decreased cilia beating frequency, although without cilia dyskinetic beating patterns or decreased protein expression. As parents disclaimed respiratory complaints, the ciliary beat frequency findings were inferred to be due to incomplete variant influence or to secondary inflammation.

## Conclusions

We report two brothers with PCD carrying in heterozygosity two *DNAH5* variants, the truncated pathogenic variant c.4237C > T p.(Gln1413*) and the missense variant of unknown significance c.5290 T > C p.(Ser1764Pro). Sanger sequencing of the variants in parents confirmed the presence of compound heterozygosity. Expression studies, including family members, and predictive structural *DNAH5* protein analysis, enabled the reclassification as pathogenic of the *DNAH5* c.5290 T > C p.(Ser1764Pro) missense variant and pathogenicity confirmation of the truncated *DNAH5* variant c.4237C > T p.(Gln1413*), which will favor inclusion in routine genetic diagnostics. Taking into account the present findings and those from other patients (from the literature), intriguing phenotype features could be uncovered, where, although with decreased cilia motility and presence of an ODA defect, exactly the same variants yield distinct PCD phenotypes, fertile/infertile and absence of laterality defects/situs inversus, suggesting a possible gene interaction effect [50–52]. This supporting data may thus contribute to a better understanding of the relationships between phenotypes-genotypes in PCD.

## Supplementary Information

Below is the link to the electronic supplementary material.

ESM 1 (19.8 MB DOCX)

ESM 2 (1.88 MB DOCX)

ESM 3 (34.1 MB DOCX)

## Data Availability

The data that support the findings of this study are available from the corresponding author, upon reasonable request.
