# Supplementary material for: Structural and functional characterization of DNAH5 variants in a Portuguese family with primary ciliary dyskinesia
Source: J Assist Reprod Genet. 2026 Jun 19;43(8):2641–57. doi: 10.1007/s10815-026-03930-1 (PMC13598022; doi:10.1007/s10815-026-03930-1)
Supplement: Supplementary file 1 [file 10815_2026_3930_MOESM1_ESM.docx]

*****

*****

**N**

*****

**A**

**N**

*****

**Supplementary Figure 1**. **A**. Displacement or misalignment of basal bodies (*) in relation to longitudinally well orientated cilia axonemes (n).

*****

*****

*****

*****

**N**

**N**

**N**

*****

**B**

*****

**Supplementary Figure 1**. **B**. Displacement or misalignment of basal bodies (*) in relation to longitudinally well orientated cilia axonemes (n).

**N**

**N**

**N**

*****

*****

**N**

*****

**C**

**Supplementary Figure 1**. **C**. Displacement or misalignment of basal bodies (*) in relation to longitudinally well orientated cilia axonemes (n).
