# Supplementary material for: Structural and functional characterization of DNAH5 variants in a Portuguese family with primary ciliary dyskinesia
Source: J Assist Reprod Genet. 2026 Jun 19;43(8):2641–57. doi: 10.1007/s10815-026-03930-1 (PMC13598022; doi:10.1007/s10815-026-03930-1)
Supplement: Supplementary file 2 [file 10815_2026_3930_MOESM2_ESM.docx]

**Supplementary Figure 2**. Quantitative transmission electron microscopy determination of the ciliary beat axis and ciliary deviation. **A**. **Step 1**. Chosen of a cross-section that shows complete ciliary membrane and intact axoneme structure.

**Supplementary Figure 2**. Quantitative transmission electron microscopy determination of the ciliary beat axis and ciliary deviation. **B**. **Step 2**. A blue line is drawn parallel to the central microtubules in each ciliary axoneme cross section.

*

**Supplementary Figure 2**. Quantitative transmission electron microscopy determination of the ciliary beat axis and ciliary deviation. **C**. **Step 3**. Based on the main orientation of the blue lines (red arrow), a reference red line was then drawn. This red line is perpendicular to the blue lines and passes between the two microtubules of the central pair of a chosen cilium (*). The angle of each blue line to the reference line is then calculated. Angles are then subtracted from their mean. The mean of the subtracted angles is equal or lower than zero. The standard deviation (SD) of the subtracted angles corresponds to the ciliary beat axis and ciliary deviation [32].
