# Supplementary material for: Structural and functional characterization of DNAH5 variants in a Portuguese family with primary ciliary dyskinesia
Source: J Assist Reprod Genet. 2026 Jun 19;43(8):2641–57. doi: 10.1007/s10815-026-03930-1 (PMC13598022; doi:10.1007/s10815-026-03930-1)
Supplement: Supplementary file 3 [file 10815_2026_3930_MOESM3_ESM.docx]

| **Supplementary Table 1**. Genotypes of *DNAH5* variants attaining the N-terminal/Linker domains with the proband variants | | | | | | | |
| --- | --- | --- | --- | --- | --- | --- | --- |
| **Reference** | **Age (years)**  **/Sex** | **Variant**  **classification** | **Variant** | **Protein**  **change** | **Zygosity** | **Molecular**  **consequence** | **Exon** |
| Present | 44/M | P | c.4237C>T | p.(Gln1413*) | HTZ | nonsense | 27 |
|  |  | VUS->P | c.5290T>C | p.(Ser1764Pro) | HTZ | missense | 33 |
|  |  |  |  |  |  |  |  |
| *Kim et al, 2014 [43] | 6/F | P | c.4237C>T | p.(Gln1413*) | HTZ | nonsense | 27 |
|  |  |  |  |  |  |  |  |
|  | 14/M | P | c.4237C>T | p.(Gln1413*) | HTZ | nonsense | 27 |
|  |  |  |  |  |  |  |  |
| *Marshall et al, 2015 [44] | 6/F | P | c.4237C>T | p.(Gln1413*) | HTZ | nonsense | 27 |
|  |  |  |  |  |  |  |  |
|  | 14/M | P | c.4237C>T | p.(Gln1413*) | HTZ | nonsense | 27 |
|  |  |  |  |  |  |  |  |
| Wheway et al, 2021 [47] | P16 | VUS | c.5290T>C | p.(Ser1764Pro) | HTZ | missense | 33 |
|  |  | VUS | c.1799T>C | p.(Ile600Thr) | HTZ | missense | 14 |
|  |  | P/LP | c.6304C>T | p.(Arg2102Cys) | HTZ | missense | 39 |
|  |  |  |  |  |  |  |  |
| Alexandru et al, 2022 [46] | P | VUS | c.5290T>C | p.(Ser1764Pro) | HZ | missense | 33 |
|  |  |  |  |  |  |  |  |
| Tinoco et al, 2023 [45] | 8/M | P | c.4237C>T | p.(Gln1413*) | HTZ | nonsense | 27 |
|  |  | VUS | c.5290T>C | p.(Ser1764Pro) | HTZ | missense | 33 |
|  |  |  |  |  |  |  |  |
|  | 30/M | P | c.4237C>T | p.(Gln1413*) | HTZ | nonsense | 27 |
|  |  | VUS | c.5157C>T | p.(Phe1719=) | HTZ | missense | 32 |
|  |  |  |  |  |  |  |  |
| Santos et al, 2025 [48] | 17/F | VUS | c.5290T>C | p.(Ser1764Pro) | HTZ | missense | 33 |
|  |  | P | C.1852C>T | p.(Arg618*) | HTZ | nonsense | 14 |

| **Supplementary Table 2**. Phenotypes of *DNAH5* variants attaining the N-terminal/Linker domains with the proband variants | | | | | | | | | | | | | |
| --- | --- | --- | --- | --- | --- | --- | --- | --- | --- | --- | --- | --- | --- |
| **Reference** | **Age (years)**  **/Sex** | **Variant** | **Consanguinity** | **Situs** | **nNO**  **(nL/min)** | **PICADAR** | **CBF**  **(Hz)** | **CBP**  **(%IM)** | **TEM** | **IF** | **Fertility** | **Segregation** |  |
| Present | 44/M | c.4237C>T | N | N | 30 | 3 | 0,66 | 62,5 | ODA | sev D | Fertile | P |  |
|  |  | c.5290T>C |  |  |  |  |  |  |  |  |  | M |  |
|  |  |  |  |  |  |  |  |  |  |  |  |  |  |
| *Kim et al, 2014 [43] | 6/F | c.4237C>T | N | SA | 22 |  |  |  | ODA |  |  |  |  |
|  |  |  |  |  |  |  |  |  |  |  |  |  |  |
|  | 14/M | c.4237C>T | N | SS | 8,9 |  |  |  | ODA |  |  |  |  |
|  |  |  |  |  |  |  |  |  |  |  |  |  |  |
| *Marshall et al, 2015 [44] | 6/F | c.4237C>T | N | SA | 22 |  |  |  | ODA |  |  |  |  |
|  |  |  |  |  |  |  |  |  |  |  |  |  |  |
|  | 14/M | c.4237C>T | N | SS | 8,9 |  |  |  | ODA |  |  |  |  |
|  |  |  |  |  |  |  |  |  |  |  |  |  |  |
| Wheway et al, 2021 [47] | P16 | c.5290T>C |  | SI |  | 8 | 0 | IM | ODA |  |  | P |  |
|  |  | c.1799T>C |  |  |  |  |  |  |  |  |  | P |  |
|  |  | c.6304C>T |  |  |  |  |  |  |  |  |  | M |  |
|  |  |  |  |  |  |  |  |  |  |  |  |  |  |
| Alexandru et al, 2022 [46] | P | c.5290T>C |  |  |  |  |  |  | ODA |  |  |  |  |
|  |  |  |  |  |  |  |  |  |  |  |  |  |  |
| Tinoco et al, 2023 [45] | 8/M | c.4237C>T |  | SIT |  | 7 |  |  |  |  | OAT |  |  |
|  |  | c.5290T>C |  |  |  |  |  |  |  |  |  |  |  |
|  |  |  |  |  |  |  |  |  |  |  |  |  |  |
|  | 30/M | c.4237C>T |  | N | low | 6 | 4,81 | DA/IM | ODA |  |  |  |  |
|  |  | c.5157C>T |  |  |  |  |  |  |  |  |  |  |  |
|  |  |  |  |  |  |  |  |  |  |  |  |  |  |
| Santos et al, 2025 [48] | 17/F | c.5290T>C |  | SIT |  |  |  |  |  |  |  |  |  |
|  |  | C.1852C>T |  |  |  |  |  |  |  |  |  |  |  |

Accession Number: NM_001369.3(DNAH5)

Gene: DNAH5

Domain: N-terminal/Linker

*Patients of Kim et al, 2014 and of Marshall et al, 2015 are the same.

Age/Sex: M: male; F: female; P16: Patient16 (no age given); P: patient (no age given)

Variant classification: P: pathogenic; LP: likely pathogenic; VUS: variant of unknown significance

Zygosity: HZ: homozygosity; HTZ: heterozygosity

Consanguinity: N: no

Situs: laterality defects: SA: situs ambiguous; SS: situs solitus; SI: situs inversus; SIT: situs inversus totalis; N: no

nNO: nitric oxide levels

CBF: cilia beat frequency

CBP: cilia beat pattern: DA: decreased amplitude; IM: immotility

TEM: transmission electron microscopy defect: ODA: outer dynein arm defect

IF: immunofluorescence: sev D: severely decreased

Fertility: OAT: oligoasthenoteratozoospermia

Segregation: P: paternal; M: maternal
